# Effects of tumor necrosis factor inhibitors and tocilizumab on the glycosylated hemoglobin levels in patients with rheumatoid arthritis; an observational study

**DOI:** 10.1371/journal.pone.0196368

**Published:** 2018-04-25

**Authors:** Yukimi Otsuka, Chikako Kiyohara, Yusuke Kashiwado, Takuya Sawabe, Shuji Nagano, Yasutaka Kimoto, Masahiro Ayano, Hiroki Mitoma, Mitsuteru Akahoshi, Yojiro Arinobu, Hiroaki Niiro, Koichi Akashi, Takahiko Horiuchi

**Affiliations:** 1 Department of Medicine and Biosystemic Science, Kyushu University Graduate School of Medical Sciences, Fukuoka, Japan; 2 Department of Internal Medicine and Clinical Immunology, Kyushu University Beppu Hospital, Beppu, Japan; 3 Department of Preventive Medicine, Kyushu University Graduate School of Medical Sciences, Fukuoka, Japan; 4 Division of Rheumatology, Hiroshima Red Cross Hospital & Atomic-bomb Survivors Hospital, Hiroshima, Japan; 5 Center for Rheumatology, Iizuka Hospital, Iizuka, Japan; 6 Department of Medical Education, Kyushu University Graduate School of Medical Sciences, Fukuoka, Japan; Keio University, JAPAN

## Abstract

Rheumatoid arthritis (RA) and diabetes mellitus (DM) are associated with inflammation. We tried to investigate the influence of tumor necrosis factor inhibitors (TNFi) and tocilizumab (TCZ) on the glucose metabolism of RA patients. RA patients in whom treatment with TNFi or TCZ was initiated from 2008 to 2015 were studied based on their medical records. We analyzed patients whose glycosylated hemoglobin (HbA1c) levels were measured both before and 3 months after the initiation of these biologic agents. The association between HbA1c reduction and the treatment was evaluated. From 971 cases treated with these biologic agents, 221 cases whose medical records of HbA1c were available, were included (TNFi, n = 154; TCZ, n = 67). Both the TNFi and TCZ groups had significantly lower HbA1c values at 1 month and 3 months after the initiation of treatment (TNFi, p<0.001; TCZ, p<0.001). Although the pretreatment HbA1c values did not differ (TNFi, 6.2%; TCZ, 6.2%; p = 0.532), the 3-month treatment HbA1c values were lower (TNFi, 6.1%; TCZ, 5.8%; p = 0.010) and the changes in HbA1c (ΔHbA1c) were greater (TNFi, 0.1%; TCZ, 0.4%; p<0.001) in the TCZ group. The reduction of HbA1c—defined by the achievement of a ΔHbA1c of ≥0.5%—was associated with baseline diagnosis of diabetes mellitus, baseline diabetes treatment, hospitalization, medical change during the observation period, and TCZ. In the multivariate logistic regression analysis, TCZ was associated with the reduction of HbA1c in comparison to TNFi (adjusted OR = 5.59, 95% CI = 2.56–12.2; p<0.001). The HbA1c levels in RA patients were significantly lower after the initiation of TNFi or TCZ. Our study suggests that TCZ decreases the HbA1c levels in RA patients to a greater extent than TNFi.

## Introduction

Rheumatoid arthritis (RA) is an inflammatory disease that is localized in joints and which also causes systemic complications. The rates of cardiovascular (CV) morbidity and mortality are increased in RA patients [1, 2], which shortens their life-span. The risk of CV disease (CVD) in RA is intricately affected by the RA activity, medications, and other CVD risk factors [3–6]. One of the most important factors is diabetes mellitus (DM) [7, 8], the pathogenesis of which is also associated with inflammation and immune system [9]. Elevation in circulating levels of acute-phase proteins, cytokines and chemokines has been reported in type 2 DM patients [10–13]. The circulating levels of C-reactive protein (CRP), interleukin-1β (IL-1β) and IL-6 are elevated in type 2 DM even before the onset of diabetes [11, 14]. Indeed, insulin resistance in RA patients is increased, which is associated with accelerated coronary atherosclerosis [15]. Given that RA and DM are associated with inflammation, these two diseases may affect each other.

Biologic agents that target specific cytokines or molecules, and which may improve glucose metabolism, have recently been applied in the treatment of rheumatic diseases. The targeting IL-1β—in order to treat DM—has been evaluated in several clinical trials [16]. Some of the studies reported the improvement of the glycosylated hemoglobin (HbA1c) level and the insulin secretion rate in type 2 DM patients, while others showed no significant changes. Some studies have shown that the administration of tumor necrosis factor (TNF)-α inhibitors (TNFi), such as infliximab (IFX), etanercept (ETN) and adalimumab (ADA) improves insulin resistance in patients with rheumatic disease [17–21], while others have reported contradictory findings [22, 23]. One retrospective cohort study shows that TNFi and methotrexate (MTX) therapy versus MTX therapy alone was slightly—but not significantly—associated with changes in HbA1c levels among psoriasis and RA patients [24]. An anti-IL-6 receptor antibody, tocilizumab (TCZ), which has been approved for the treatment of RA in Japan since 2008, is reported to improve insulin sensitivity in patients without DM (n = 11) during rheumatoid disease treatment [25]. Of great interest, HbA1c improvement was observed after 6 months of TCZ treatment for RA in both diabetic (n = 10) and non-diabetic (n = 29) patients [26]. However, the effects of steroid tapering as well as other possible confounding factors were not considered in these previous studies; thus, it was difficult to draw any definite conclusions on the relationship between anti-inflammatory drugs and the improvement of glucose intolerance. Moreover, no reports have investigated differences in the glucose metabolism effects of the different biologic agents. It would be more definite to evaluate the influence of different biologic agents on the on the HbA1c level, than to compare biologic agents to non-biologic agents such as MTX, since the backgrounds and clinical courses of patients treated with biologic agents would be similar.

In order to clarify whether biologic agents could improve the glucose metabolism in RA patients and whether there is any difference between biologic agents, we sought to investigate the associations between TNFi or TCZ treatment for RA and the reduction of the HbA1c levels in a retrospective observational study.

## Methods

### Patients

RA patients who were diagnosed according to the RA classification standard by the American College of Rheumatology (ACR) in 1987 or the ACR and European League Against Rheumatism (EULAR) in 2010 were identified from four institutions in the western part of Japan (Kyushu University hospital, Fukuoka; Kyushu University Beppu Hospital, Beppu; Hiroshima Red Cross Hospital & Atomic-bomb Survivors Hospital, Hiroshima; Aso Iizuka Hospital, Iizuka); two are university hospitals and the others are large medical centers affiliated to Kyushu University. We identified all of the RA patients in whom treatment with TNFi or TCZ was initiated and continued for more than 3 months between January 2008 and December 2015 from their medical records. The TNFi included IFX, ETN, ADA, golimumab (GLM), and certolizumab pegol (CZP). Patients who switched biologic agents or in whom treatment with the same biologic agent was restarted after more than 6 months of cessation were included as different cases. We made no distinctions regarding the route of administration (intravenous or subcutaneous). Patients were exposed based on the typical RA infusion intervals and doses. We searched for patients whose pretreatment HbA1c values available. Patients in whom the HbA1c levels were not measured at 3 months after the initiation of TNFi or TCZ were excluded. From a total of 971 potentially eligible cases in which treatment with TNFi or TCZ was initiated, we excluded those without pretreatment HbA1c value (n = 444) and those who lacked HbA1c value at 3 months (n = 306). Kyushu University Ethics Review Board and the review boards of each participating institution approved the study protocol and privacy precautions (28–204). All data were fully anonymized before we accessed them and the ethics committees waived the requirement for informed consent.

### Main outcome variable

The primary outcome was the change in HbA1c from pretreatment values to the 3-month treatment value. We defined ΔHbA1c as the pretreatment HbA1c value–the 3-month treatment value. We defined a reduction of HbA1c as the achievement of a ΔHbA1c decrease of ≥0.5%. Considering that the coefficient of variance is 2–3% in the measurement of HbA1 [27], a 0.5% decrease in the HbA1c value would be large enough to be identified as a reduction when the pre-treatment HbA1c values between 4.0% and 14.9%.

### Study factors

We evaluated the following demographic values and the potential confounding factors at the initiation of TNFi or TCZ from medical records: sex, age, body mass index (BMI), duration of RA, positivity of rheumatoid factor (RF), joint damage (Steinbrocker stage), daily dysfunction (Steinbrocker class), a history of using other biologic agents, DM diagnosis at baseline, medication for RA and DM, DAS28-CRP levels, and the modified Health Assessment Questionnaire Disability Index (mHAQ-DI). The criteria of DM diagnosis in this study was defined as the pretreatment HbA1c value ≥6.5% or use of any diabetes drugs at baseline.

### Other variables

We defined the observation period as the 30th day before the initiation of treatment to the 90th day after the initiation of treatment with TNFi or TCZ. The following factors, which would influence the change in the HbA1c value during the observation period were identified: a history of hospitalization for more than 2 days, medical changes with regard to oral glucocorticoid (GC) or antidiabetic agents, the best DAS28- CRP response, improvement of mHAQ-DI, and the changes of hemoglobin levels. We searched for patients with a history of hospitalization for more than 2 days during the observation period, as patients for whom the initiation of biologic treatment is planned are often in hospital to screen for infections and neoplasms or to evaluate the effects and side effects of the biologic agents. Hospitalization might lead to improved blood sugar control, as patients receive calorie controlled meals and appropriate medications. The tapering of oral GC doses due to the administration of biologic agents might also reduce the HbA1c value. We defined the tightening of diabetes treatment as adding another type of medication, changing the type of medication, or increasing the dose of anti-diabetes agents or insulin. Disease activity was categorized as follows: DAS28 remission (DAS28-CRP < 2.3), low disease activity (2.3 ≤ DAS28-CRP < 2.7), moderate disease activity (2.7 ≤ DAS28-CRP ≤ 4.1), and high disease activity (DAS28-CRP > 4.1) [28]. The DAS28-CRP response was defined according to the EULAR response criteria as none, moderate, or good [29]. We identified the lowest DAS28-CRP value during the period from the 30th day to the 105th (90+15) day after the initiation of biologic treatment and evaluated the best DAS28-CRP response. Physical function was measured using the mHAQ-DI [30]. The improvement of mHAQ-DI score was defined as a decrease of ≥0.22 units in mHAQ scores in the context of minimum clinically important differences of 0.22 [31]. The hemoglobin levels increased in RA patients during the treatment with TCZ, which is reported to be greater than other biologic agents [32]. The changes of hemoglobin levels might affect the HbA1c values of RA patients.

### Procedures

The measurements that were performed closest the initiation of TNFi or TCZ were selected as the pretreatment values (a maximum of 30 days from the day of initiation). The measurements closest to the 90th day after the initiation of TNFi or TCZ were selected as the 3-month treatment values (extension period: a maximum of ±15 days from the 90th day). Similarly, the measurements performed closest to the 30th day after the day of initiation were used as the 1-month treatment values (extension period: a maximum of ±15 days from the 30th day). Other data and the DAS28-CRP values were collected on the same day as the HbA1c measurement or the day closest to the day of HbA1c measurement (within the same extension period). The HbA1c values and the hemoglobin levels were derived from routine clinical laboratory practice. In Japan, HbA1c was evaluated according to the HbA1c (JDS) values until March 2012, which are lower than the National Glycohemoglobin Standardization Program (NGSP) values. In this study, the HbA1c (NGSP) (%) value was determined by adding 0.4% to the HbA1c (JDS) (%) value [27], in order to standardize the values. Thus, all of the HbA1c values are shown as NGSP values.

### Statistical analysis

The baseline characteristics of the patients that were treated with TNFi were compared with those that were treated with TCZ. We used the two-sample t-test or the Wilcoxon–Mann–Whitney test for continuous variables and the χ2 test for categorical variables. The changes in the HbA1c values of each group were assessed using the Friedman test and the Wilcoxon signed rank test with Bonferroni correction. An unconditional logistic regression analysis was used to compute the odds ratios (ORs) and corresponding 95% confidence intervals (CIs), with adjustments for several covariates that were found to be associated with the reduction of HbA1c (age, sex, DM diagnosis at baseline, use of any diabetes drugs at baseline, hospitalization for more than 2 days, medical change [reduction of oral glucocorticoids and the tightening of diabetes treatment], and the initiation of TNFi or TCZ treatment). To assure the results, we additionally performed the similar analyses, changing the definition of the reduction of HbA1c by the achievement of a ΔHbA1c of ≥0.4% or 0.6%. The statistical analyses were performed using the JMP Pro Version 11 software program (SAS Institute, Cary, North Carolina, USA) and the STATA version 14.2 software program (STATA Corporation, College Station, TX, USA). All of the p values were two-sided and p values of <0.05 were considered to indicate statistical significance.

## Results

The baseline characteristics of the 221 patients (TNFi, n = 154; TCZ, n = 67) are shown in Table 1. The TNFi that was administered in the TNFi group included: IFX, n = 25; ETN, n = 41; ADA, n = 33; GLM, n = 44; and CZP, n = 11. The patients in the TNFi group were older (67.8 years vs 64.2 years, p = 0.036) and the frequency of RF positive was less (67.4% vs 82.1%, p = 0.022). There was no significant difference in the frequency of DM diagnosis and the use of any diabetes drugs at baseline. The use of tacrolimus (TAC) and oral glucocorticoid (GC), which are both known to have unfavorable effects on the glucose metabolism, were similar in both groups, as was the dose of oral GC. In the TNFi group, methotrexate (MTX) was more frequently used and the doses were higher (68.8% vs 40.3%, p<0.001; and 5.5 mg/week vs 3.2 mg/week, p<0.001; respectively), and less patients had previously been treated with any biologic agent (27.9% vs 49.3%, p = 0.002) in comparison to the TCZ group. Although there were missing data, the DAS28-CRP values before treatment were slightly lower in the TNFi group (4.1 vs 4.4, p = 0.095). On the other hand, the mHAQ-DI scores tended to be similar at baseline.

**Table 1:** The baseline characteristics of all the study subjects, overall and according to biologic agents.

	All (n = 221)	TNF inhibitors (n = 154)	n*	Tocilizumab (n = 67)	n*	p-value
**Basic and clinical**						
Female sex, n (%)	68.8	70.1	0	65.7	0	0.513
Age (years), mean (SD)	66.7 (11.6)	67.8 (11.0)	0	64.2 (12.7)	0	0.036
RA duration (years), mean (SD)	10.5 (12.5)	10.4 (12.2)	0	10.9 (13.4)	0	0.767
RA duration ≤ 2 years, n (%)	27.1	27.9	0	25.4	0	0.694
Stage I or II, n (%)	56.6	56.5	0	56.7	0	0.976
Class I or II, n (%)	77.8	79.2	0	74.6	0	0.454
RF positive, n (%)	72.0	67.4	7	82.1	0	0.022
BMI (kg/m^2^), mean (SD)	23.1 (4.3)	23.3 (4.5)	10	22.8 (3.8)	2	0.484
BMI categories, n (%)			10		2	0.564
<18.5	9.1	10.4		6.2		
18.5–24.9	67.5	64.6		73.8		
25.0–29.9	15.8	16.7		13.8		
≥30.0	7.6	8.3		6.2		
DM diagnosis, n (%)	49.5	49.0	0	50.8	0	0.814
**Medication**						
MTX, n (%)	60.2	68.8	0	40.3	0	<0.001
MTX (mg/week), mean (SD)	4.8 (4.6)	5.5 (4.5)	0	3.2 (4.5)	0	<0.001
TAC, n (%)	9.5	9.7	0	9.0	0	0.854
Oral GC, n (%)	58.4	60.4	0	53.7	0	0.357
Oral GC (mg/day), mean (SD)	4.0 (4.4)	3.8 (3.9)	0	4.4 (5.4)	0	0.338
Any diabetes drugs, n (%)	37.1	36.4	0	38.3	0	0.730
Any previous biologic treatment (ever), n (%)	34.4	27.9	0	49.3	0	0.002
**Data from** **pre-treatment**						
DAS28-CRP, mean (SD)	4.2 (1.3)	4.1 (1.3)	34	4.4 (1.2)	11	0.095
High disease activity, n (%)	51.7	51.7	34	51.8	11	0.988
mHAQ-DI, median (IQR)	0.81 (0.25–1.25)	0.75 (0.25–1.38)	53	0.88 (0.25–1.25)	20	0.942

BMI, body mass index; CI, confidence interval; Class, Steinbrocker class; CRP, C-reactive protein; DM, diabetes mellitus; GC, glucocorticoid; IQR, interquartile range; mHAQ-DI, the modified Health Assessment Questionnaire Disability Index; MTX, methotrexate; RA, rheumatoid arthritis; RF, rheumatoid factor; SD, standard deviation; Stage, Steinbrocker stage; TAC, tacrolimus.

* Number of missing values

**Fig 1:**
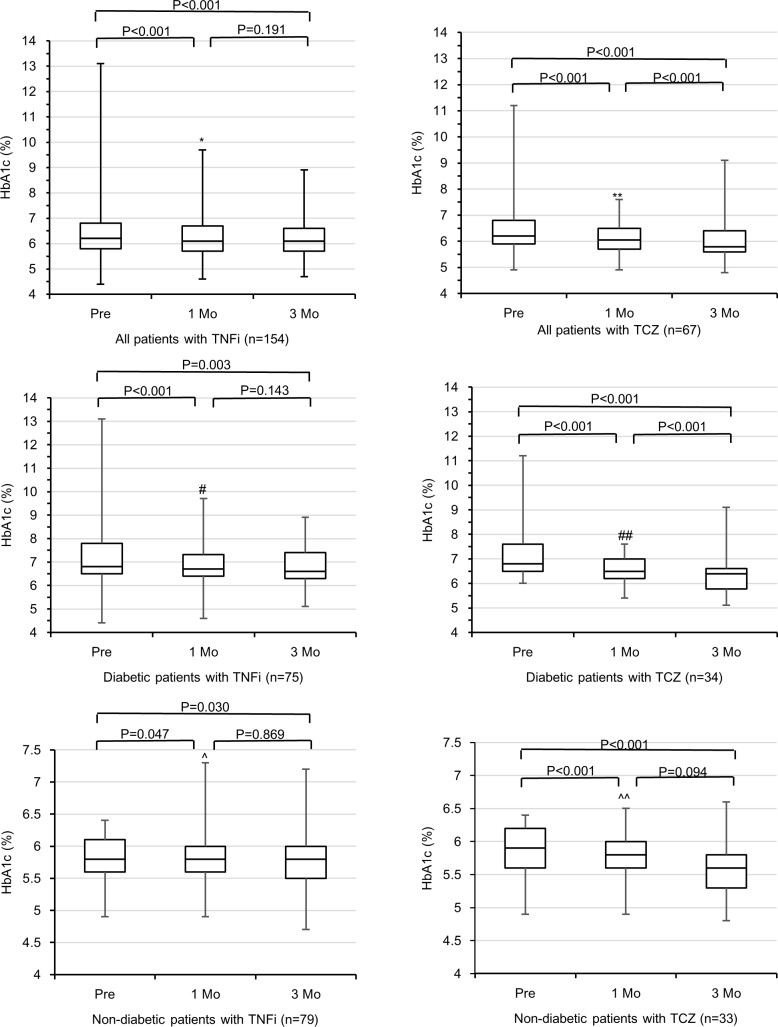
Effects of TNF inhibitors (TNFi) and tocilizumab (TCZ) on glycosylated hemoglobin (HbA1c) in 1 month and 3 months after initiation of biologic agents. Boxes indicate interquartile range and horizontal bars represent median values. Whiskers indicate most extreme points. Changes in HbA1c were evaluated by the Friedman test, which were significant in all the six groups (p<0.001). Subsequently, each HbA1c values were compared by the Wilcoxon signed rank test. The significance level was adjusted as 0.017 by Bonferroni method. *Number of missing value is 25. ** Number of missing value is 13. # Number of missing value is 13. ## Number of missing value is 7. ^ Number of missing value is 12. ^^ Number of missing value is 6.

The changes in the HbA1c values were evaluated in each biological agent group; then the subgroup analysis was performed in which the patients were classified into a diabetic group and a non-diabetic group depending on DM diagnosis at baseline (Fig 1). After the initiation of treatment, the HbA1c values were significantly changed in both the TNFi and TCZ groups (TNFi, p<0.001; TCZ, p<0.001). The HbA1c values at both 1 month and 3 months decreased significantly after the initiation of TNFi or TCZ (TNFi, p<0.001; TCZ, p<0.001). In the subgroup analysis, a Friedman test revealed significant changes in the HbA1c values of each group regardless of the presence of DM. However, only the non-diabetic group with TNFi had no significant change by the Wilcoxon signed rank test after adjusting the significance level as 0.017 by Bonferroni method.

Changes in the HbA1c value and other medical conditions in the observation period are shown in Table 2. There were no significant differences in factors that potentially affected the HbA1c change, such as a reduction of the oral glucocorticoid dose and the tightening of diabetes treatment. The best DAS28-CRP response differed according to the biologic agents and the responses seemed to be better in the TCZ group. However, the improvement of mHAQ-DI scores tended to be similar in both groups. As previously reported, the hemoglobin levels after 3 months treatment were greater in the TCZ group than TNFi (12.4g/dL vs 13.0g/dL, p = 0.010). Although the pretreatment HbA1c values did not differ to a statistically significant extent (6.2% vs 6.2%, p = 0.532), the 3-month treatment values in the TCZ group were significantly lower (6.1% vs 5.8%, p = 0.010). Likewise, in the TCZ group, the decrease in the ΔHbA1c was greater (0.1% vs 0.4%, p<0.001) and patients more frequently achieved a ΔHbA1c of ≥0.5% in comparison to the TNFi group (19.5% vs 47.8%, p<0.001). Similar tendencies were observed in the subgroup analysis, suggesting that the initiation of TCZ tended to lower the HbA1c values to a greater extent than TNFi. On the other hand, the increase in hemoglobin levels did not contribute to the changes in the HbA1c values among all patients, the TNFi group, and the TCZ group (S1 Table).

**Table 2:** The changes in the HbA1c values and other medical conditions in the observation period.

	All (n = 221)	TNF inhibitors (n = 154)	n*	Tocilizumab (n = 67)	n*	p-value
Hospitalization for more than 2 days, n (%)	39.8	37.7	0	44.8	0	0.322
**Medical change**						
Reduction of oral GC dose, n (%)	29.9	27.3	0	35.8	0	0.206
Tightening of diabetes treatment, n (%)	14.5	14.3	0	14.9	0	0.901
**Data at 3 months**						
DAS28-CRP, mean (SD)	2.4 (1.1)	2.5 (1.1)	42	2.1 (1.0)	15	0.011
mHAQ-DI, median (IQR)	0.38 (0–1.0)	0.38 (0–1.0)	52	0.25 (0–0.9)	22	0.212
**Best DAS28-CRP response, n (%)**			49		13	<0.001
no response	18.9	23.8		9.2		
moderate response	33.3	40.0		20.4		
good response	47.8	36.2		70.4		
**Improvement of mHAQ-DI**						
mHAQ-DI MCID of ≥0.22, n (%)	46.2	44.0	63	51.2	26	0.439
**Hemoglobin (g/dL), mean (SD)**						
pre-treatment	12.0 (1.6)	12.1 (1.6)	0	11.9 (1.6)	0	0.340
after I month	12.4 (1.6)	12.3 (1.6)	0	12.5 (1.6)	0	0.347
after 3 months	12.6 (1.6)	12.4 (1.6)	0	13.0 (1.5)	0	0.010
**Hemoglobin A1c (%) in all patients, median (IQR)**						
pre-treatment	6.2 (5.8–6.8)	6.2 (5.8–6.8)	0	6.2 (5.9–6.8)	0	0.532
after 1 month	6.1 (5.7–6.6)	6.1 (5.7–6.7)	25	6.05 (5.7–6.5)	13	0.374
after 3 months	6.0 (5.7–6.5)	6.1 (5.7–6.6)	0	5.8 (5.6–6.4)	0	0.010
ΔHbA1c (%), median (IQR)	0.2 (-0.1–0.5)	0.1 (-0.1–0.4)	0	0.4 (0.1–0.8)	0	<0.001
ΔHbA1c≧0.5%, n (%)	28.1	19.5	0	47.8	0	<0.001
**Hemoglobin A1c (%) in diabetic patients, median (IQR)**						
pre-treatment	6.8 (6.5–7.6)	6.8 (6.5–7.8)	0	6.8 (6.5–7.4)	0	0.781
after 1 month	6.6 (6.35–7.2)	6.7 (6.4–7.33)	13	6.5 (6.2–7.0)	7	0.092
after 3 months	6.5 (6.2–7.1)	6.6 (6.3–7.4)	0	6.4 (5.78–6.6)	0	0.005
ΔHbA1c (%), median (IQR)	0.3 (-0.1–0.9)	0.2 (-0.2–0.9)	0	0.7 (0.3–0.9)	0	0.004
ΔHbA1c≧0.5%, n (%)	45.0	34.7	0	67.7	0	0.001
**Hemoglobin A1c (%) in non-diabetic patients, median (IQR)**						
pre-treatment	5.9 (5.6–6.1)	5.8 (5.6–6.1)	0	5.9 (5.6–6.2)	0	0.493
after 1 month	5.8 (5.6–6.0)	5.8 (5.6–6.0)	12	5.8 (5.6–6.0)	6	0.603
after 3 months	5.7 (5.5–6.0)	5.8 (5.5–6.0)	0	5.6 (5.3–5.8)	0	0.036
ΔHbA1c (%), median (IQR)	0.1 (-0.1–0.3)	0.1 (-0.1–0.2)	0	0.2 (0.05–0.5)	0	0.004
ΔHbA1c≧0.5%, n (%)	11.6	5.1	0	27.3	0	0.002

CI, confidence interval; CRP, C-reactive protein; GC, glucocorticoid; HbA1c, glycosylated hemoglobin; IQR, interquartile range; MCID, minimum clinically important differences; mHAQ-DI, the modified Health Assessment Questionnaire Disability Index; SD, standard deviation.

* Number of missing values

A univariate logistic regression analysis was performed to evaluate the factors that contributed to the reduction of the HbA1c values (Table 3). A reduction of the HbA1c was associated with sex, the baseline diagnosis of DM (crude OR = 6.22, 95% CI = 3.12–12.4; p<0.001), the baseline use of any diabetes drugs (crude OR = 4.57, 95% CI = 2.45–8.53; p<0.001), hospitalization for more than 2 days (crude OR = 2.36, 95% CI = 1.30–4.29; p = 0.005), medical change in the reduction of the oral GC dose (crude OR = 2.62, 95% CI = 1.41–4.87; p = 0.002) or the tightening of diabetes treatment (crude OR = 11.9, 95% CI = 4.97–28.6; p<0.001), and TCZ treatment (crude OR = 3.78, 95% CI = 2.03–7.05; p<0.001). The other factors for which significant differences were observed between the two groups including the changes of hemoglobin levels did not contribute to the reduction of HbA1c.

**Table 3:** The results of the univariate logistic regression analysis of factors associated with the reduction of HbA1c.

Variables	OR	95% CI	p-value
Sex, female vs. male	0.42	0.23–0.78	0.006
Age, 65≥ vs. 65<	1.18	0.64–2.19	0.592
RA duration ≤2 years	0.72	0.36–1.42	0.342
Stage, I+ II vs. III + IV	0.99	0.55–1.80	0.984
Class, I + II vs. III + IV	1.26	0.61–2.62	0.530
RF positive	1.28	0.65–2.52	0.479
BMI in categories			
<18.5 kg/m2	0.67	0.21–2.15	0.505
18.5–24.9 kg/m2	1.0 (reference)		
25.0–29.9 kg/m2	1.44	0.65–3.21	0.368
≥30.0 kg/m2	0.84	0.26–2.76	0.776
DM diagnosis at baseline	6.22	3.12–12.4	<0.001
Baseline medication			
MTX	0.56	0.31–1.01	0.055
TAC	1.32	0.51–3.44	0.572
Oral GC, 1≥ vs. 0	1.74	0.94–3.22	0.079
Any diabetes drugs	4.57	2.45–8.53	<0.001
Any previous biologic treatment (ever)	1.18	0.64–2.17	0.597
High disease activity at baseline	1.29	0.66–2.51	0.460
Hospitalization for more than 2 days	2.36	1.30–4.29	0.005
Medical change			
Reduction of oral GC dose	2.62	1.41–4.87	0.002
Tightening of diabetes treatment	11.9	4.97–28.6	<0.001
Best DAS28-CRP response	n = 159		
no response	1.0 (reference)		
moderate response	0.96	0.33–2.78	0.943
good response	1.43	0.54–3.79	0.477
Improvement of mHAQ-DI	1.59	0.69–3.63	0.275
Change of hemoglobin, increase vs. no change or decrease	1.1	0.56–2.14	0.779
TCZ vs. TNFi	3.78	2.03–7.05	<0.001

BMI, body mass index; CI, confidence interval; Class, Steinbrocker class; CRP, C-reactive protein; DM, diabetes mellitus; GC, glucocorticoid; HbA1c, glycosylated hemoglobin; mHAQ-DI, the modified Health Assessment Questionnaire Disability Index; MTX, methotrexate; OR, odds ratio; RA, rheumatoid arthritis; RF, rheumatoid factor; Stage, Steinbrocker stage; TAC, tacrolimus; TCZ, tocilizumab; TNFi, tumor necrosis factor inhibitors.

The results of a multivariate logistic regression analysis of factors associated with the reduction of HbA1c are shown in Table 4. The age- and sex-adjusted ORs were statistically significant for all variables in Table 4. After mutual adjustment for age, sex, and all variables in Table 4—with the exception of the tightening of diabetes treatment (multivariate adjusted OR = 6.77, 95% CI = 2.35–19.5; p<0.001)—only one factor was significantly associated with the reduction of the HbA1c value. TCZ treatment (multivariate adjusted OR = 5.59, 95% CI = 2.56–12.2; p<0.001, in comparison to TNFi treatment) was significantly associated with the reduction of the HbA1c value. The additional analyses performed in changing the definition of the reduction of HbA1c are shown in S2, S3, S4 and S5 Tables. Whether we define the reduction of HbA1c as the achievement of a ΔHbA1c of ≥0.4% or 0.6%, TCZ treatment was significantly associated with the reduction of the HbA1c value compared with TNFi treatment.

**Table 4:** The results of the multivariate logistic regression analysis of factors associated with the reduction of HbA1c.

Variables	Adjusted OR* (95% CI)	p-value	Adjusted OR** (95% CI)	p-value
DM diagnosis at baseline	5.64 (2.79–11.4)	<0.001	3.13 (0.99–9.88)	0.051
Any diabetes drugs at baseline	4.40 (2.32–8.34)	<0.001	1.32 (0.45–3.86)	0.610
Hospitalization for more than 2 days	2.55 (1.38–4.74)	0.003	1.28 (0.59–2.79)	0.528
Reduction of oral GC dose	2.60 (1.38–4.88)	0.003	1.71 (0.80–3.67)	0.168
Tightening of diabetes treatment	11.8 (4.82–28.9)	<0.001	6.77 (2.35–19.5)	<0.001
TCZ vs. TNFi	3.99 (2.09–7.61)	<0.001	5.59 (2.56–12.2)	<0.001

CI, confidence interval; CRP, C-reactive protein; DM, diabetes mellitus; GC, glucocorticoid; HbA1c, glycosylated hemoglobin; OR, odds ratio; TCZ, tocilizumab; TNFi, tumor necrosis factor inhibitors.

*Adjusted for age and sex.

** Mutually adjusted for age, sex, and all variables in Table 4.

## Discussion

Our observational study showed a significant reduction in the HbA1c values at both one month and three months after the initiation of TNFi or TCZ treatment (Fig 1). The influence of biologic agents on the glucose metabolism of RA patients is so complicated that it is difficult to interpret the changes in the HbA1c values as direct effects of anti-inflammatory agents. Treatment with biologic agents would result in lower RA disease activity, leading to a lower dose of GC and better physical activities. Patients may exercise more and medication adherence may be improved, inducing better insulin sensitivity and blood sugar control. In addition, the HbA1c values are altered by anemia [33], though the effect of which is inconsistent [34, 35]. Thus, it might not be accurate to conclude that the biologic agents directly improved the HbA1c value. It would be reasonable, however, to compare the effects of different biologic agents on the HbA1c level in order to minimize the confounding factors that could influence the glucose metabolism. This is the first report to compare the influence of biological agents on the glucose metabolism during RA treatment.

The data from our study indicates that, in comparison to the initiation of TNFi treatment, the initiation of TCZ treatment was more strongly associated with the reduction of the HbA1c level (Table 4). It is of note that the reduction of HbA1c was not dependent on the improvement of anemia (S1 Table, Table 3). This result may be due to difference in roles of IL-6 and TNF-α in glucose metabolism. TCZ is a humanized antibody, which blocks both membrane-bound IL-6 receptors and soluble IL-6 receptor [36]. IL-6 is known to have paradoxical role in metabolic processes [37]. Circulating IL-6 is elevated due to obesity, resulting in hepatic insulin resistance [38], which implies that IL-6 has unfavorable effects on glucose metabolism. In contrast, there are several reports of possible favorable effects. IL-6 from skeletal muscle stimulates glucagon-like peptide-1 from the gut and pancreas [39]. Global IL-6 knockout mice develop mature-onset obesity [40]. These reports indicate the favorable effects of IL-6 on the metabolic processes. One of the reasons for the controversial role of IL-6 is that IL-6 has complex signaling pathways. It is currently thought that the unfavorable effects of IL-6 on the metabolic processes may occur due to ‘trans-signaling’ [37]. Trans-signaling is conducted by soluble forms of IL-6 receptors (IL-6R). In trans-signaling, IL-6 binds to soluble IL-6R, which enables the signaling of this complex in cells expressing glycoprotein 130 (gp130) but not IL-6R on their membrane [37]. The mechanism to accelerate the favorable impact of IL-6 on metabolic processes is currently being investigated—these studies may lead to a new treatment method for metabolic syndrome and type 2 DM.

The effects of TCZ on the metabolic processes are also complicated. It is widely known that modest elevations of low-density lipoprotein-cholesterol (LDL-C), high density lipoprotein-cholesterol (HDL-C), and triglycerides are observed in RA patients who are treated with TCZ [41], which is a concern due to the increased risk of CVD. In MEASURE [42], a randomized placebo-controlled study to investigate the effects of TCZ on surrogates of vascular risk, TCZ induced the elevations of LDL-C, but HDL particles were changed towards an anti-inflammatory composition. The effects of biologic agents on CVD have been reported. Treatment with TNFi for >16.1 months was associated with a decreased risk for CVD in RA [43]. The multi-database population-based cohort study showed no evidence of an increased cardiovascular risk among RA patients who switched from a different biologic drug or tofacitinib to TCZ versus to TNFi [44]. ENTRACTE study, a randomized controlled trial comparing CV safety of TCZ vs ETN in RA patients with risk factors for CVD, reported that lipid changes induced by TCZ do not translate into an increased risk of CVD in RA patients [45]. Indeed, genome-wide association studies [46, 47] suggest that IL-6R signaling is likely to have great effects on the vascular risk and blocking IL-6R may be a therapeutic approach to prevent coronary heart disease. Sarilumab, an anti–IL-6R human monoclonal antibody, also seems to reduce HbA1c values in diabetic and nondiabetic patients with RA compared with placebo, which is supporting favorable effects of blocking IL-6R signaling on glucose metabolism [48]. Thus far, only a few reports have investigated the impact of TCZ on the glucose metabolism and insulin sensitivity [25, 26, 49]. Although all of these studies reported the favorable effects of TCZ on HbA1c or insulin resistance in RA patients, the number of patients participating in these studies was small. More clinical information is required to reveal whether TCZ has favorable effects on the glucose metabolism.

Our study is associated with several limitations. Since this was a retrospective study, the possibility of overlooking unmeasured confounding factors cannot be excluded. We included patients who had previously received other biologic agents. Studies with biologic-naïve patients may be required to confirm this report. Some data were missing from the medical records, including HbA1c values. This might have led to a selection bias. Since most of DM patients in our institutions underwent HbA1c measurement once a month, however, the impact of possible bias is likely to be limited. Finally, the number of cases was not large enough, especially for evaluating the differences among the TNFi; thus, all 5 types of TNFi (IFX, ETN, ADA, GLM, and CZP) were regarded as the same group. In fact, the effects of complete anti-TNF antibodies (IFX, ADA, and GLM) on TNF-producing cells differ from the effects of ETN and CZP [50, 51].

As biologic agents have become a new therapeutic choice for the treatment of RA, the prognosis of disease activities and the quality of life of RA patients have improved dramatically. The mortality of RA patients would also be ameliorated by the use of biologic agents. RABBIT, a cohort study from Germany, showed that TNFi, rituximab, and other biologic agents seemed to reduce the risk of mortality in RA patients in comparison to patients receiving MTX [52]. However, it has not been revealed whether there are differences between biologic agents. Our study suggests that TCZ decreases the HbA1c levels to a greater extent than TNFi in RA patients with relatively high HbA1c values. Further studies are warranted to interpret the effects of biologic agents on the glucose metabolism.

## Supporting information

S1 Table The changes in the HbA1c values depending on the changes of hemoglobin levels in the observation period. Hb, hemoglobin; HbA1c, glycosylated hemoglobin; IQR, interquartile range; TCZ, tocilizumab; TNFi, tumor necrosis factor inhibitors.
* Number of missing values
(DOCX)

S2 Table The results of the univariate logistic regression analysis of factors associated with the reduction of HbA1c defined by the achievement of a ΔHbA1c of ≥0.4%. BMI, body mass index; CI, confidence interval; Class, Steinbrocker class; CRP, C-reactive protein; DM, diabetes mellitus; GC, glucocorticoid; HbA1c, glycosylated hemoglobin; mHAQ-DI, the modified Health Assessment Questionnaire Disability Index; MTX, methotrexate; OR, odds ratio; RA, rheumatoid arthritis; RF, rheumatoid factor; Stage, Steinbrocker stage; TAC, tacrolimus; TCZ, tocilizumab; TNFi, tumor necrosis factor inhibitors.
(DOCX)

S3 Table The results of the multivariate logistic regression analysis of factors associated with the reduction of HbA1c defined by the achievement of a ΔHbA1c of ≥0.4%. CI, confidence interval; DM, diabetes mellitus; GC, glucocorticoid; HbA1c, glycosylated hemoglobin; MTX, methotrexate; OR, odds ratio; TCZ, tocilizumab; TNFi, tumor necrosis factor inhibitors.
*Adjusted for age and sex.
** Mutually adjusted for age, sex, and all variables in S3 Table.
(DOCX)

S4 Table The results of the univariate logistic regression analysis of factors associated with the reduction of HbA1c defined by the achievement of a ΔHbA1c of ≥0.6%. BMI, body mass index; CI, confidence interval; Class, Steinbrocker class; CRP, C-reactive protein; DM, diabetes mellitus; GC, glucocorticoid; HbA1c, glycosylated hemoglobin; mHAQ-DI, the modified Health Assessment Questionnaire Disability Index; MTX, methotrexate; OR, odds ratio; RA, rheumatoid arthritis; RF, rheumatoid factor; Stage, Steinbrocker stage; TAC, tacrolimus; TCZ, tocilizumab; TNFi, tumor necrosis factor inhibitors.
(DOCX)

S5 Table The results of the multivariate logistic regression analysis of factors associated with the reduction of HbA1c defined by the achievement of a ΔHbA1c of ≥0.6%. CI, confidence interval; DM, diabetes mellitus; GC, glucocorticoid; HbA1c, glycosylated hemoglobin; OR, odds ratio; TCZ, tocilizumab; TNFi, tumor necrosis factor inhibitors.
*Adjusted for age and sex.
** Mutually adjusted for age, sex, and all variables in S5 Table.
(DOCX)
