# Supplementary material for: Effects of tumor necrosis factor inhibitors and tocilizumab on the glycosylated hemoglobin levels in patients with rheumatoid arthritis; an observational study
Source: PLoS One. 2018 Apr 25;13(4):e0196368. doi: 10.1371/journal.pone.0196368 (PMC5918963; doi:10.1371/journal.pone.0196368)
Supplement: S1 Table — Hb, hemoglobin; HbA1c, glycosylated hemoglobin; IQR, interquartile range; TCZ, tocilizumab; TNFi, tumor necrosis factor inhibitors. * Number of missing values (DOCX) [file pone.0196368.s001.docx]

**S1 Table. The changes in the HbA1c values depending on the changes of hemoglobin levels in the observation period.**

|  | No change or decrease in Hb levels  (n = 60) | n* | Increase in Hb levels  (n = 161) | n* | p-value |
| --- | --- | --- | --- | --- | --- |
| **Hemoglobin A1c (%) in all patients, median (IQR)** |  |  |  |  |  |
| pre-treatment | 6.2  (5.8-6.7) | 0 | 6.2  (5.9-6.9) | 0 | 0.797 |
| after 1 month | 6.3  (5.9-6.6) | 10 | 6.1  (5.7-6.6) | 28 | 0.201 |
| after 3 months | 6.1  (5.7-6.6) | 0 | 6.0  (5.7-6.5) | 0 | 0.620 |
| ΔHbA1c (%),  median (IQR) | 0.2  (-0.1-0.5) | 0 | 0.2  (-0.1-0.5) | 0 | 0.917 |
| ΔHbA1c≧0.5%, n (%) | 26.7 | 0 | 28.6 | 0 | 0.779 |
| **Hemoglobin A1c (%) in the TNFi group, median (IQR)** |  |  |  |  |  |
| pre-treatment | 6.2  (5.8-6.6) | 0 | 6.3  (5.8-6.9) | 0 | 0.747 |
| after 1 month | 6.2  (5.8-6.6) | 8 | 6.1  (5.7-6.7) | 17 | 0.612 |
| after 3 months | 6.1  (5.7-6.6) | 0 | 6.1  (5.7-6.7) | 0 | 0.795 |
| ΔHbA1c (%),  median (IQR) | 0.1  (-0.1-0.4) | 0 | 0.1  (-0.2-0.4) | 0 | 0.391 |
| ΔHbA1c≧0.5%, n (%) | 23.1 | 0 | 17.7 | 0 | 0.421 |
| **Hemoglobin A1c (%) in the TCZ group, median (IQR)** |  |  |  |  |  |
| pre-treatment | 6.6  (6.0-7.3) | 0 | 6.2  (5.9-6.8) | 0 | 0.535 |
| after 1 month | 6.4  (6.1-7.0) | 2 | 6.0  (5.7-6.5) | 11 | 0.087 |
| after 3 months | 5.9  (5.6-6.4) | 0 | 5.8  (5.6-6.4) | 0 | 0.734 |
| ΔHbA1c (%),  median (IQR) | 0.4  (0.1-0.9) | 0 | 0.4  (0.1-0.8) | 0 | 0.727 |
| ΔHbA1c≧0.5%, n (%) | 50.0 | 0 | 47.5 | 0 | 0.893 |

Hb, hemoglobin; HbA1c, glycosylated hemoglobin; IQR, interquartile range; TCZ, tocilizumab; TNFi, tumor necrosis factor inhibitors.

* Number of missing values
