# Supplementary material for: Effects of tumor necrosis factor inhibitors and tocilizumab on the glycosylated hemoglobin levels in patients with rheumatoid arthritis; an observational study
Source: PLoS One. 2018 Apr 25;13(4):e0196368. doi: 10.1371/journal.pone.0196368 (PMC5918963; doi:10.1371/journal.pone.0196368)
Supplement: S2 Table — BMI, body mass index; CI, confidence interval; Class, Steinbrocker class; CRP, C-reactive protein; DM, diabetes mellitus; GC, glucocorticoid; HbA1c, glycosylated hemoglobin; mHAQ-DI, the modified Health Assessment Questionnaire Disability Index; MTX, methotrexate; OR, odds ratio; RA, rheumatoid arthritis; RF, rheumatoid factor; Stage, Steinbrocker stage; TAC, tacrolimus; TCZ, tocilizumab; TNFi, tumor necrosis factor inhibitors. (DOCX) [file pone.0196368.s002.docx]

**S2 Table.** The results of the univariate logistic regression analysis of factors associated with the reduction of HbA1c defined by the achievement of a ΔHbA1c of ≥0.4%

| Variables | OR | 95% CI | p-value |
| --- | --- | --- | --- |
| Sex, female vs. male | 0.50 | 0.28-0.90 | 0.021 |
| Age, 65≥ vs. 65< | 1.25 | 0.70-2.23 | 0.450 |
| RA duration ≤2 years | 0.80 | 0.45-1.51 | 0.491 |
| Stage, I+ II vs. III + IV | 1.16 | 0.67-2.04 | 0.593 |
| Class, I + II vs. III + IV | 1.48 | 0.74-2.96 | 0.266 |
| RF positive | 1.41 | 0.74-2.68 | 0.294 |
| BMI in categories |  |  |  |
| <18.5 kg/m2 | 0.49 | 0.15-1.54 | 0.220 |
| 18.5-24.9 kg/m2 | 1.0 (reference) |  |  |
| 25.0-29.9 kg/m2 | 1.52 | 0.70-3.27 | 0.287 |
| ≥30.0 kg/m2 | 1.09 | 0.37-3.18 | 0.872 |
| DM diagnosis at baseline | 3.60 | 2.00-6.48 | <0.001 |
| Baseline medication |  |  |  |
| MTX | 0.57 | 0.32-0.99 | 0.047 |
| TAC | 1.42 | 0.57-3.54 | 0.448 |
| Oral GC, 1≥ vs. 0 | 1.33 | 0.76-2.34 | 0.322 |
| Any diabetes drugs | 2.53 | 1.43-4.48 | 0.001 |
| Any previous biologic treatment (ever) | 1.02 | 0.57-1.81 | 0.958 |
| High disease activity at baseline | 1.34 | 0.72-2.50 | 0.353 |
| Hospitalization for more than 2 days | 1.92 | 1.09-3.37 | 0.023 |
| Medical change |  |  |  |
| Reduction of oral GC dose | 2.44 | 1.35-4.43 | 0.003 |
| Tightening of diabetes treatment | 7.50 | 3.17-17.7 | <0.001 |
| Best DAS28-CRP response | n=102 |  |  |
| no response | 1.0 (reference) |  |  |
| moderate response | 0.72 | 0.27-1.90 | 0.505 |
| good response | 1.30 | 0.54-3.17 | 0.557 |
| Improvement of mHAQ-DI | 1.01 | 0.48-2.11 | 0.984 |
| Change of hemoglobin,  increase vs. no change or decrease | 1.25 | 0.66-2.35 | 0.491 |
| TCZ vs. TNFi | 3.40 | 1.87-6.19 | <0.001 |

BMI, body mass index; CI, confidence interval; Class, Steinbrocker class; CRP, C-reactive protein; DM, diabetes mellitus; GC, glucocorticoid; HbA1c, glycosylated hemoglobin; mHAQ-DI, the modified Health Assessment Questionnaire Disability Index; MTX, methotrexate; OR, odds ratio; RA, rheumatoid arthritis; RF, rheumatoid factor; Stage, Steinbrocker stage; TAC, tacrolimus; TCZ, tocilizumab; TNFi, tumor necrosis factor inhibitors.
