# Supplementary material for: Effects of tumor necrosis factor inhibitors and tocilizumab on the glycosylated hemoglobin levels in patients with rheumatoid arthritis; an observational study
Source: PLoS One. 2018 Apr 25;13(4):e0196368. doi: 10.1371/journal.pone.0196368 (PMC5918963; doi:10.1371/journal.pone.0196368)
Supplement: S3 Table — CI, confidence interval; DM, diabetes mellitus; GC, glucocorticoid; HbA1c, glycosylated hemoglobin; MTX, methotrexate; OR, odds ratio; TCZ, tocilizumab; TNFi, tumor necrosis factor inhibitors. *Adjusted for age and sex. ** Mutually adjusted for age, sex, and all variables in S3 Table. (DOCX) [file pone.0196368.s003.docx]

**S3 Table.** The results of the multivariate logistic regression analysis of factors associated with the reduction of HbA1c defined by the achievement of a ΔHbA1c of ≥0.4%

| Variables | Adjusted OR* (95% CI) | p-value | Adjusted OR**  (95% CI) | p-value |
| --- | --- | --- | --- | --- |
| DM diagnosis at baseline | 3.28 (1.80 – 5.97) | <0.001 | 3.10 (1.14 – 8.48) | 0.027 |
| Any diabetes drugs  at baseline | 2.37 (1.32 – 4.25) | 0.004 | 0.64 (0.24 – 1.73) | 0.376 |
| MTX at baseline | 0.58 (0.33 – 1.03) | 0.064 | 1.11 (0.54 – 2.29) | 0.771 |
| Hospitalization  for more than 2 days | 2.00 (1.13 – 3.56) | 0.018 | 1.15 (0.56 – 2.36) | 0.694 |
| Reduction of oral GC dose | 2.41 (1.32 – 4.41) | 0.004 | 1.78 (0.89 – 3.55) | 0.101 |
| Tightening of  diabetes treatment | 7.24 (3.04 – 17.3) | <0.001 | 5.07 (1.83 – 14.0) | <0.001 |
| TCZ vs. TNFi | 3.56 (1.92 – 6.59) | <0.001 | 4.21 (2.08 – 8.49) | <0.001 |

CI, confidence interval; DM, diabetes mellitus; GC, glucocorticoid; HbA1c, glycosylated hemoglobin; MTX, methotrexate; OR, odds ratio; TCZ, tocilizumab; TNFi, tumor necrosis factor inhibitors.

*Adjusted for age and sex.

** Mutually adjusted for age, sex, and all variables in S3 Table.
