# Supplementary material for: Effects of tumor necrosis factor inhibitors and tocilizumab on the glycosylated hemoglobin levels in patients with rheumatoid arthritis; an observational study
Source: PLoS One. 2018 Apr 25;13(4):e0196368. doi: 10.1371/journal.pone.0196368 (PMC5918963; doi:10.1371/journal.pone.0196368)
Supplement: S4 Table — BMI, body mass index; CI, confidence interval; Class, Steinbrocker class; CRP, C-reactive protein; DM, diabetes mellitus; GC, glucocorticoid; HbA1c, glycosylated hemoglobin; mHAQ-DI, the modified Health Assessment Questionnaire Disability Index; MTX, methotrexate; OR, odds ratio; RA, rheumatoid arthritis; RF, rheumatoid factor; Stage, Steinbrocker stage; TAC, tacrolimus; TCZ, tocilizumab; TNFi, tumor necrosis factor inhibitors. (DOCX) [file pone.0196368.s004.docx]

**S4 Table.** The results of the univariate logistic regression analysis of factors associated with the reduction of HbA1c defined by the achievement of a ΔHbA1c of ≥0.6%

| Variables | OR | 95% CI | p-value |
| --- | --- | --- | --- |
| Sex, female vs. male | 0.46 | 0.24-0.89 | 0.021 |
| Age, 65≥ vs. 65< | 1.12 | 0.57-2.17 | 0.747 |
| RA duration ≤2 years | 0.73 | 0.34-1.53 | 0.403 |
| Stage, I+ II vs. III + IV | 1.03 | 0.54-1.96 | 0.926 |
| Class, I + II vs. III + IV | 1.35 | 0.60-3.01 | 0.469 |
| RF positive | 1.22 | 0.59-2.54 | 0.595 |
| BMI in categories |  |  |  |
| <18.5 kg/m2 | 0.40 | 0.09-1.83 | 0.238 |
| 18.5-24.9 kg/m2 | 1.0 (reference) |  |  |
| 25.0-29.9 kg/m2 | 1.48 | 0.64-3.43 | 0.360 |
| ≥30.0 kg/m2 | 1.14 | 0.34-3.76 | 0.835 |
| DM diagnosis at baseline | 9.40 | 3.99-22.2 | <0.001 |
| Baseline medication |  |  |  |
| MTX | 0.62 | 0.33-1.17 | 0.139 |
| TAC | 1.11 | 0.38-3.19 | 0.849 |
| Oral GC, 1≥ vs. 0 | 1.84 | 0.93-3.62 | 0.079 |
| Any diabetes drugs | 5.86 | 2.93-11.7 | <0.001 |
| Any previous biologic treatment (ever) | 0.91 | 0.46-1.78 | 0.772 |
| High disease activity at baseline | 1.21 | 0.58-2.54 | 0.604 |
| Hospitalization for more than 2 days | 2.23 | 1.17-4.25 | 0.014 |
| Medical change |  |  |  |
| Reduction of oral GC dose | 2.97 | 1.54-5.75 | 0.001 |
| Tightening of diabetes treatment | 13.2 | 5.63-30.9 | <0.001 |
| Best DAS28-CRP response | n=102 |  |  |
| no response | 1.0 (reference) |  |  |
| moderate response | 1.31 | 0.41-4.21 | 0.651 |
| good response | 1.23 | 0.40-3.75 | 0.716 |
| Improvement of mHAQ-DI | 1.07 | 0.42-2.72 | 0.888 |
| Change of hemoglobin,  increase vs. no change or decrease | 1.04 | 0.51-2.13 | 0.912 |
| TCZ vs. TNFi | 2.88 | 1.49-5.56 | 0.002 |

BMI, body mass index; CI, confidence interval; Class, Steinbrocker class; CRP, C-reactive protein; DM, diabetes mellitus; GC, glucocorticoid; HbA1c, glycosylated hemoglobin; mHAQ-DI, the modified Health Assessment Questionnaire Disability Index; MTX, methotrexate; OR, odds ratio; RA, rheumatoid arthritis; RF, rheumatoid factor; Stage, Steinbrocker stage; TAC, tacrolimus; TCZ, tocilizumab; TNFi, tumor necrosis factor inhibitors.
