# Supplementary material for: Effects of tumor necrosis factor inhibitors and tocilizumab on the glycosylated hemoglobin levels in patients with rheumatoid arthritis; an observational study
Source: PLoS One. 2018 Apr 25;13(4):e0196368. doi: 10.1371/journal.pone.0196368 (PMC5918963; doi:10.1371/journal.pone.0196368)
Supplement: S5 Table — CI, confidence interval; DM, diabetes mellitus; GC, glucocorticoid; HbA1c, glycosylated hemoglobin; OR, odds ratio; TCZ, tocilizumab; TNFi, tumor necrosis factor inhibitors. *Adjusted for age and sex. ** Mutually adjusted for age, sex, and all variables in S5 Table. (DOCX) [file pone.0196368.s005.docx]

**S5 Table.** The results of the multivariate logistic regression analysis of factors associated with the reduction of HbA1c defined by the achievement of a ΔHbA1c of ≥0.6%

| Variables | Adjusted OR* (95% CI) | p-value | Adjusted OR**  (95% CI) | p-value |
| --- | --- | --- | --- | --- |
| DM diagnosis at baseline | 8.80 (3.69 – 21.0) | <0.001 | 4.13 (1.15 – 14.8) | 0.029 |
| Any diabetes drugs  at baseline | 5.74 (2.83 – 11.7) | <0.001 | 1.43 (0.48 – 4.28) | 0.524 |
| Hospitalization  for more than 2 days | 2.38 (1.23 – 4.60) | 0.010 | 1.03 (0.44 – 2.42) | 0.942 |
| Reduction of oral GC dose | 2.93 (1.50 – 5.73) | 0.002 | 1.99 (0.89 – 4.45) | 0.096 |
| Tightening of  diabetes treatment | 13.0 (5.48 – 30.9) | <0.001 | 6.95 (2.45 – 19.7) | <0.001 |
| TCZ vs. TNFi | 2.93 (1.50 – 5.75) | 0.002 | 4.06 (1.76 – 9.36) | 0.001 |

CI, confidence interval; DM, diabetes mellitus; GC, glucocorticoid; HbA1c, glycosylated hemoglobin; OR, odds ratio; TCZ, tocilizumab; TNFi, tumor necrosis factor inhibitors.

*Adjusted for age and sex.

** Mutually adjusted for age, sex, and all variables in S5 Table.
